# Grounded Theory and Social Psychology Approach to Investigating the Formation of Construction Workers' Unsafe Behaviour

**DOI:** 10.1155/2022/3581563

**Published:** 2022-05-18

**Authors:** Yu Han, Xuezheng Li, Zhida Feng, Ruoyu Jin, Joseph Kangwa, Obas John Ebohon

**Affiliations:** ^1^Faculty of Civil Engineering and Mechanics, Jiangsu University, 301 Xuefu Road, Zhenjiang 212013, Jiangsu, China; ^2^Faculty of Management, Jiangsu University, 301 Xuefu Road, Zhenjiang 212013, Jiangsu, China; ^3^School of Built Environment and Architecture, London South Bank University, 103 Borough Rd, London SE10AA, UK; ^4^School of Architecture and Built Environment, University of Wolverhampton, Wolverhampton, UK

## Abstract

There have been limited studies analyzing the causes of construction workers' unsafe behaviour from the social psychology perspective. Based on a Grounded Theory approach, this study first identified and defined seven coded categories related to workers' dangerous behaviour on construction sites. The original qualitative data were obtained from individual site interviews conducted with 35 construction professionals. These main categories were found connected to workers' status of safety awareness and sense of danger, which affected the type of unsafe behaviours, i.e., proactive, passive, or reactive behaviour. By further integrating social cognitive psychology theories into workers' behavioural decision-making process, the formation mechanism framework and diagram were developed to describe construction workers' unsafe behaviours based on the dynamic process of balancing the individual desires and perceived safety risks. This study advances the body of knowledge in construction safety behavioural management by performing in-depth theoretical analysis regarding workers' internal desires, activated by external scenarios and intervened by a personal safety cognition system, which could result in different motivations and various behavioural outcomes. It is argued that safety cognition serves as a mediated moderation system affecting behavioural performance. Practical suggestions on developing a proper safety management system incorporating safety education in guiding construction workers' site behaviours are presented.

## 1. Introduction

Construction is generally recognized as a risky industry with high injuries and accidents [1–2]. Besides accidents/incidents and other quantitative measurements (e.g., injury rate), reactive indicators for safety performance and proactive measures have also been developed in construction, such as behaviour-based safety and safety climate/culture [3, 4]. Existing studies (e.g., [5–7]) focusing on these proactive measurements have commonly adopted the questionnaire survey approach. Potential drawbacks of conducting the questionnaire survey method include some questions being incorrectly completed, questions being misunderstood, requiring follow-up in-depth research, and being unsuitable for investigating long and complex issues [8]. In addition, the multiscale data collected through a questionnaire survey may not be sufficient to depict construction workers' subtle mental status and psychological state when conducting unsafe behaviours. Construction site workers before, during, and right after making a behavioural decision could present a dynamic and mixed internal mechanism influenced by external scenarios (e.g., the project's tight schedule). Likewise, an advanced workflow originates from workers' inner desires, which may be activated by an external scenario and mediated by personal safety cognition, resulting in safety behavioural decisions.

As the alternative approach, qualitative studies (e.g., semistructured interviews) are also adopted in construction safety research, followed by qualitative analysis to define categories related to a specific safety issue, for example, causes of fall accidents from roofs [9]. More alternative analysis approaches from other fields, such as Grounded Theory from social studies, have been applied in the construction field. So far, there have been limited studies [10, 11] that have applied Grounded Theory to construction safety. The rationale of using Grounded Theory in this study to explore the causes of workers' unsafe behaviour is not concurrent with the methodology from prior studies of construction safety. These issues, as mentioned earlier, are mainly due to the desire to link dangerous behaviour to construction sites to a social psychological definition. Furthermore, the desire to articulate the mechanism of workers' behavioural decision formulation process from the perspective of human psychology and what drives the tenants for such desires and motivations is yet another objective. Research questions in social psychology, such as by what processes attitudes towards an object affect behaviour towards the object [12], can be applied in construction safety behaviour to provide a theoretical guide in safety management.

Aiming to address the above issues, this study was designed to answer the following research questions: (1) what are the internal and external causes of construction workers' unsafe behaviour and (2) how would human psychological desires and motivations lead to different behavioural outcomes? This study addresses the research scenarios in construction workers' unsafe behaviours depicting the unique psychological mechanism of behavioural decisions. An alternative method called Grounded Theory is adopted for qualitative analysis. Therefore, this study is seen to contribute to the in-depth theory of the formation of workers' unsafe behaviour by linking workers' behaviour to their safety cognition, internal desire, and the ultimate behavioural outcome. The study provides both theoretical and practical guides for effective construction safety management. Theoretically, it postulates a framework of how internal human desires, insinuated by the mediation and moderation effects of safety cognition, could lead to different behavioural decisions and outcomes; practically, this study sequences the extent to which construction workers' safety behaviours towards a safer and less risky manner can manifest.

## 2. Literature Review

### 2.1. Safety Behaviour

Safety behaviour is a critical theme in managing safety [13]. It crosses different fields such as driving [14], fire service [15], and agriculture [16]. Innovative research theories and methods are required to promote behavioural safety studies [13]. Up to 95% of workplace accidents have been caused by unsafe acts [17]. Occupational safety behaviour significantly affects workplace performance [18]. The management of safety behaviour was identified by Goh and Ayskar Ali [19] as one key challenge in improving construction safety management. [20] described the intervention process to correct unsafe behaviours by measuring and comparing the frequency, duration, and rate of behaviours before and after the intervention, aiming to change dangerous behaviours. The behaviour-based safety (BBS) could enhance safety performance by monitoring preestablished safety behaviours in construction [21]. However, when adopting behaviour-based management in construction, Lingard and Rowlinson [22] found the behaviour-based limitation and suggested that safe behaviour could only be achieved when a basic safety infrastructure is in place. Further, Eckenfelder [23] criticized BBS for being time-consuming and costly, as safety investment would interact with the construction workforce to affect safety performance [24]. Nevertheless, Cooper [25] showed positive outcomes of applying BBS in improving safety performance. Similarly, the empirical study conducted by Choudhry [26] showed that BBS could be used in any country's culture based on effective communication on-site and committed management. Critical success factors for BBS have been identified, including employee engagement, satisfaction with safety training, and trust relationships among workers [20]. These factors affecting safety behaviour, such as work engagement and management commitment [27], could be important indicators in the safety climate framework.

### 2.2. Safety Climate

In the dynamic construction safety management system, Guo et al. [28] suggested considering the interrelationships among factors within the system. One factor within the management system is defined as a safety climate within the workforce. Attitudes towards safety are considered one of the main indicators of safety climate [5]. Safety climate can be measured by workers' safety perceptions [29], and it reflects workers' perception of the role of safety in their jobs [30]. In the safety climate study conducted by Li et al. [7] in China's construction industry, workers' self-perception of safety (e.g., safety awareness), their involvement in safety (e.g., self-protection), peer interaction (e.g., communication and cooperation), safety environment, management involvement, and safety personnel support (e.g., foremen behaviour) were defined as major dimensions. Similar safety climate indicators such as risk perception, workers' perception of safety management, and management attitudes can be found in multiple other studies (e.g., [31, 32]). The effect of safety climate on safety performance has been identified in several previous studies [33, 34]. Safety climate can be multilevel and can be divided into subgroup safety climates according to workers' job position [35, 36] and other subgroup factors such as demographic features [37].

### 2.3. Safety Cognition

A safety climate forms a culture that involves knowledge transfer at the organizational level [38] and engages social cognitions [39]. Implicit memory significantly affects unconscious cognition in making judgements [40, 41]. Cognition directly influences behaviour [42]. Individual safety cognition is critical to enhancing safety performance in the construction industry [43]. Social awareness can be divided into the implicit and explicit levels [39]. The implicit social cognition reflects individuals' internal assumptions that could influence behaviour and group perceptions [44]. In the construction industry, workers' implicit safety cognition is formed based on their prior work scenarios, which further build their safety knowledge [45, 46]. The implicit safety cognition further affects workers' intuition or basic assumptions, which, together with prior scenarios and safety knowledge, affect workers' safety perceptions [39]. Safety perception constitutes the explicit social cognition [46], which, to some degree, is equated to a safety climate based on the measurement criteria [47].

## 3. Methodology

**Figure 1:**
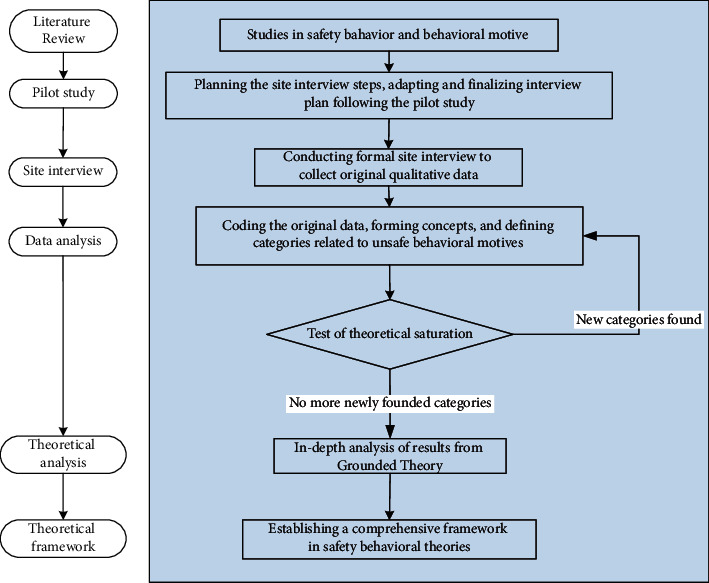
Description of the research workflow.

Investigation of research questions such as “how construction workers form their unsafe behaviours” requires in-depth research as this type of question involves complex issues. Qualitative methods (e.g., site interviews with workers) could overcome the barriers encountered in the questionnaire survey-based approach by investigating the formation of workers' unsafe behaviours. A more qualitative approach can explore insights into human beings' opinions, attitudes, experiences, and behaviours [48]. Face-to-face site interview with individual construction workers to collect qualitative data on reasons for risk-taking behaviours has been adopted in some existing studies, including Guo et al. [28] and Man et al. [11], who assumed the Grounded Theory approach. Grounded Theory aims to generate or discover a theory from data systematically obtained from social research [49]. It has been successfully adopted to understand a concept or phenomenon in different fields, such as higher education [50], housing policies [51], and healthcare [52]. The methodology adopted in this study is illustrated in Figure 1.

The data analysis method shown in Figure 1 involves the three-step Grounded Theory approach, namely, open, axial, and selective coding, as Strauss and Corbin [53, 54] advised. After capturing the causes of workers' unsafe behaviours, theoretical saturation is tested by utilising the remaining data to ensure that no more new concepts or categories would be found. The Grounded Theory is based on an explanatory approach to investigate a specific occurrence in an inductive way which favours the exact explanation of the phenomenon studied [55]. Researchers also followed other methodological guides in adopting Grounded Theory in this study, specifically: (1) early stages of data collection may involve purposeful sampling [56] as evidenced in the example of MacDonald [57]; (2) as data collection and analysis progress, e.g., concepts and categories summarized and refined from data [58], ideas, and tentative hypotheses could emerge [59]; and (3) pursing theories through data and theoretical sampling [60]. Following the interviews with workers, the qualitative data were recorded and analyzed using the intelligent voice core technological tool developed by iFlytek [61], which assisted speech recognition by analyzing the oral characteristics of human beings.

### 3.1. Design of Interview Questions

Following the review of safety behaviour-related literature, the open-ended questions for site interviews with construction workers were designed. They underwent the pilot study to ensure that these questions were valid, transparent, and easily understood. Before conducting the pilot study, the research ethics approval was gained from Jiangsu University. A consent form would be provided to each interview invitee before conducting the interview. It was ensured that no personal or company information would be stored in a database. Invitees to participate in the interview would be provided with a clear guide of the purpose of the study, and they could decide whether or not to accept the interview request. They were also allowed to withdraw or terminate the process during the interview.

Considering the generally low level of education that workers had received in China's construction industry and other facts that they tended to hide or withhold sensitive information related to their unsafe behaviours to the interviewer, these questions were designed as simple as possible to be not technical. In April 2018, before conducting the formal site interview, a pilot study involving five site workers was asked these predesigned questions to evaluate the appropriateness and effectiveness of the discussion. These five participants worked as project managers, safety staff, site technicians, crew supervisors, and steelworkers. Their age ranged from 33 to 52, with site experience varying from 9 to 31 years. The interview with each participant lasted from 18 to 57 minutes. Following the pilot study, researchers in this study found that generally, interviewees would be happy to share their experiences regarding safety issues with others when they were just bystanders not involved. However, they would be more nervous or concerned when asked of themselves as the party directly involved in safety issues, despite that they were told that the interview was confidential and anonymous. Therefore, in the finalized questions listed in Table 1, the interview with each participant was designed to start with a relaxing atmosphere in a storytelling style by sharing his or her own site experience.

The five basic questions in Table 1 were designed to be open-ended as they can be easily extended to discover more details contributing to site workers' unsafe behaviour or behavioural decision. They considered the potential psychological status of interviewees progressively. Researchers asked guidance and descriptive and summative questions by starting with the general question with the interviewee as a bystander or witness, then shifting the interviewee's role to be the party directly involved in unsafe behaviour, and finally back to the role as a bystander. Basic information related to dangerous behaviour can be obtained from answers to these questions. More insightful or detailed information could also be acquired by directing these open-ended questions.

### 3.2. Background Information of Interviewees

Echoing Cutcliffe [56] and MacDonald [57], who indicated the adoption of purposeful sampling in collecting data, this study recruited interviewees based on the critical features known to the researchers. These key features included knowledge or experience on the studied phenomenon of interest, availability and willingness to participate, and the ability to communicate experiences and opinions expressively and reflectively, which were defined by Palinkas et al. [62] for purposeful sampling. A total of 35 participants were recruited in the formal site interview conducted from May 2018 to March 2019 in the southeast coastal region of China. The reasons for recruiting participants from the eastern coastal region of China can be more in the prior studies of researchers (i.e., [37, 46]). Basically, as the economically active region of China, the east coastal region attracted labours from the rest of the country to work in the construction industry. These construction workers were also referred to as migrant workers that were considered representative of the workforce sample. The qualitative data for Grounded Theory-based analysis were based on the interview of 30 participants. The remaining five participants' answers to interview questions were used for the later theoretical saturation test described in Figure 1. The background information of the 35 interviewees is provided in Table 2. The professions of interviewees included project manager, quality inspector, general contractor's site workers, safety staff, crew supervisors, and workers from different trades. The average site experience was 16 years in the interviewee sample, and 60% of them had an education level below or at middle school. The average time lasted in each interview was around 29 minutes.

### 3.3. Interview Process

A semistructured interview was conducted individually for each participant. The interview process was designed to be interactive, allowing the mutual discussion and questions between the interviewer and the interviewee. Interviewees were encouraged to express their opinions freely. Considering that site workers usually have heavy daily duties in a relatively confined and stressful work environment, the interviews were generally conducted in the evenings after participants completed their daily tasks and were in a relaxing mental state. During each interview, the researcher started with an icebreaker to allow the interviewee to feel comfortable. The interviewer (i.e., the researcher) encouraged the interviewee to move forward with the safety behaviour-related theme by adopting the questions listed in Table 1, simultaneously enabling the interviewee to be comfortable. Following each response from the interviewee, the interviewer would then continue by asking, “what you just said is insightful. Do you have any other things to share with me?” With each interviewee's full consent and approval, the interview was voice-recorded.

## 4. Results from Grounded Theory Analysis

The categorization process involves the cyclical workflow of analyzing, inducing, and summarizing the sentences and keywords from original voice messages conveyed by the interviewees. Coding is a crucial job in the analysis and summary. It involves labelling interviewees' answers and selecting, distinguishing, and categorizing the qualitative data. Therefore, the three main steps, including open, axial, and selective coding, were conducted progressively and concurrently.

### 4.1. Open Coding Results

Open coding intends to define concepts and categories. The main objective of open coding is to disaggregate original data and reform the concepts until there is no more suitable code to replace the resulting and improved concepts. Glaser [63] suggested using the gerund format as being more ideal for exploring the concepts of Grounded Theory, for example, “saving effort” instead of “to save effort,” as shown in Table 3. In this study, the original data were analyzed on a single-word basis, with the actual words in the text labelled to define the initial factors. During the induction and summarization processes, interviewees' sentences were compared and matched, and prototypical keywords were marked. Initial concepts were extracted based on these labelled concepts and semantic meanings within sentences. Finally, all concepts with similar or consistent attributes were classified into initially coded categories. For example, in the original verbal message, “I feel it cumbersome to wear a safety belt, making me not flexible to do my job,” the keywords “cumbersome” and “not flexible” were extracted to form the initial concept of “avoiding cumbersome operation.” In another verbal message, “Guys would like to find the most convenient way to work, and it is common to find an easier way to save time,” the keywords “convenient” and “to save time” were captured. These similar keywords from other messages were found during open coding, and they were formed into the initial category defined as “saving efforts.”

The 255 messages conveyed by the 30 selected interviewees were initially obtained by adopting the iFLYTEK [61] tool. The research team performed a second round of screening by removing codes not related to construction safety. 158 messages were finalized and converted into coded concepts. Table 3 displays some of the concepts associated with typical examples from interviewees' original verbal statements.

These concepts in Table 3 are further coded into initial categories as shown in Table 4.

The open-coding results reveal that site workers who conduct unsafe behaviours may be due to their psychological needs (e.g., seeking excitement) or motivations (e.g., increasing income). It is also indicated that workers' unsafe behaviours are affected by external conditions or site scenarios. For instance, they may desire to follow their peers' behaviours, follow the demand from their team leader to work fast, or even simply help others in an emergency.

### 4.2. Axial Coding Results

Following the open coding, axial coding aims to establish a more generalized category through cluster analysis to discover internal connections between these initial categories identified in Table 4. It can be found from Tables 3 and 4 that some of the concepts are strongly correlated, for example, saving time and pursuing work efficiency. According to the behavioural intention and motivations reflected in the open coding, these initially coded categories are redefined into seven main categories listed in Table 5.

These seven main categories listed in Table 5 could be further divided into three different scenarios: (1) reducing physical work, saving time, and increasing income are motivations that workers hold to enhance their input-to-output efficiency. Workers desire to meet these personal needs. Under this scenario, they behave unsafely in a proactive way to achieve personal needs; (2) relieving stress and wishing to be part of the team are scenarios that drive workers to adjust themselves in a specialized circumstance, although it may not be their original intention to make these adjustments. They adopt unsafe behaviours to cater to the external scenario (e.g., managers' demand to work fast) in a passive manner; (3) different from the prior two scenarios, workers desire to help others, especially under emergent situations, despite their lack of safety knowledge or competency. Their unsafe behaviour manifests reactively.

### 4.3. Selective Coding Results

The principles of selective coding were defined by Strauss and Corbin [53, 54] as the process of selecting the main category, relating it to other types, and analyzing the relationships between categories. Selective coding was conducted through text analyses to identify the internal connections for each main category defined from axial coding, workers' individual safety perception, and the corresponding and resulting unsafe behaviour type. Each coded main category is listed in Table 5, with individuals' safety perception status, and the potential behavioural outcomes are described in Table 6.

It is seen that individual desire or needs, activated by one of these main categories, linked to the unique perception status, could potentially lead to unsafe behaviour. Based on the qualitative data obtained and processed from the initial steps, the safety perception status of individuals can be defined in distinct types according to their safety awareness and fear of danger. The perception status described in Table 6 indicates that safety education is imperative for individuals to be equipped with sufficient safety awareness and the correct perceptions of the ensuing danger. Their unsafe behavioural outcome can also be divided into proactive, passive, and reactive types. These various perception statuses and types of the behavioural effect can further be described as (1) the intention of saving time, reducing labour inputs, or increasing personal income, all of which have the potential to reward the respective workers themselves; moreover, behind the income-driven motivations is the devastating effect of proactive unsafe behaviours; (2) relieving stress and the desire to be part of the team could induce workers to behave unprofessionally due to the external stress or peer pressure; and (3) in a less common case, helping or rescuing others on-site under emergency also causes unsafe behaviours. This scenario can be defined as workers' reactive behaviour stimulated by unexpected stresses.

### 4.4. Test of Theoretical Saturation

Following the preliminary three-step coded analysis of qualitative data from the 30 interviewees, data from the remaining five interviewees were adopted for the theoretical saturation test. Following the same three-step analysis in Grounded Theory, no new concepts or categories were different from the previously defined categories. Therefore, it was indicated that the current coded categories and their connections, as described in Table 6, had encapsulated the significant causes and features of unsafe behaviours on construction sites comprehensively. Further discussion and in-depth theoretical analysis could then be conducted.

## 5. Discussion

### 5.1. Analytic Framework in the Formation of Safety Behavioural Decision

**Figure 2:**
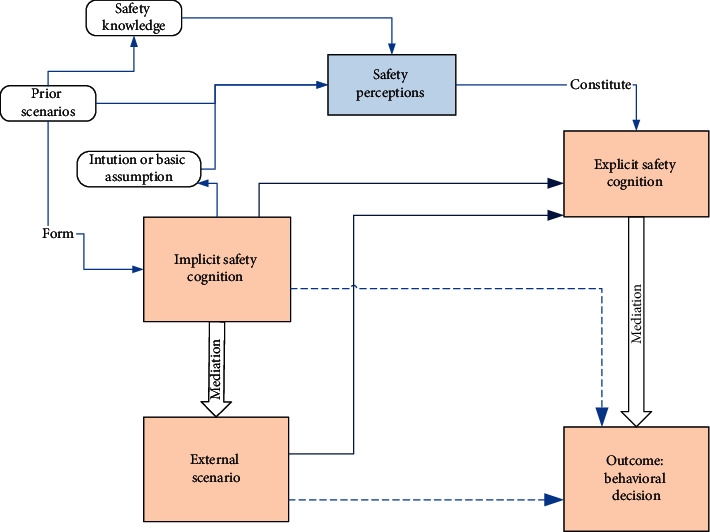
Analytic framework of mediated moderation informing construction workers' safety behavioural decision (source adapted by integrating the theories of Baron and Kenny [64] and Han et al. [46]).

Following the Grounded Theory analysis, the process from construction workers' internal desires or motivations to behavioural outcomes is analyzed by introducing the social psychology theories as demonstrated in Figure 2.

#### 5.1.1. Definition of the Analytic Framework Involving Mediated Moderation Effects in Safety Behavioural Outcomes

The framework shown in Figure 2 is considered a combination of mediation and moderation, according to Baron and Kenny [64]. The difference between a moderator (e.g., implicit safety cognition) and a mediator (e.g., explicit awareness) lies in that a moderator serves as an independent variable. Still, a mediator works as an intervening factor between an independent variable and the outcome [64]. As seen in Figure 2, workers' implicit safety cognition is at the same level as the external scenario as an independent variable that may affect the outcome, which is the worker's behavioural decision of whether to behave unsafely. The external methods can activate these coded main categories shown in Table 5, such as the desire to save effort or gain more income. According to Scheier [65] and Snyder [66], self-monitoring or self-consciousness, once becoming the moderator variable, can improve the traits and attitudes. In this study, workers' self-monitoring or self-consciousness of safety can be reflected in the implicit safety cognition as moderating variables to the external scenarios. In this dynamic framework involving multiple variables affecting the behavioural outcome, self-monitoring within the implicit safety cognition, as a moderator variable, divides workers into subgroups according to their traits, for example, by demographic factors [67], job trades [68], or experience level [37]. These subgroup factors could cause workers' safety perceptions [46].

#### 5.1.2. Extending the Social Psychology Theory into Safety Behavioural Science

**Figure 3:**
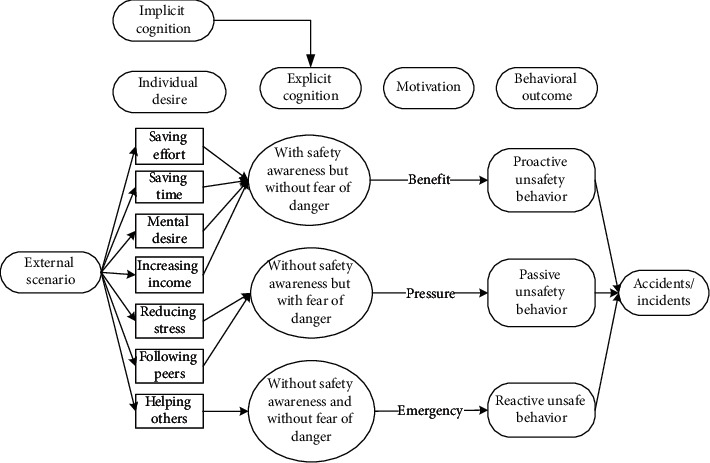
Diagram describing the formation of workers' unsafe behaviours driven by various individual desires under different explicit cognition patterns.

Applying the social phycology theory described in Baron and Kenny [64] and following the dynamic framework displayed in Figure 2, it is seen that the implicit safety cognition moderates the effects of the external scenarios in their behavioural decision, and the explicit safety cognition works as the mediator who could significantly intervene the decision. The behavioural decision could be different depending on the interacted effects of these variables. This interacted effect can be through different routes by extending the theory of Baron and Kenny [64]. The implicit cognition and the external scenario may significantly affect the behavioural decision (as seen in the dashed lines in Figure 3 from implicit safety cognition and external scenario to the outcome). The interaction of explicit cognition and external scenario indicates moderation. Explicit cognition can mediate the relation from the external scenario to the outcome, meaning that the external distraction from these aforementioned coded categories (e.g., desire to increase personal income) does not necessarily cause unsafe behaviours if the worker has the correct safety perception to resist the temptations activated by the external scenarios. If that mediation is complete, these temptations that are driven by the external site scenarios to work unsafely would not result in unsafe behaviours. Indeed, to strengthen the explicit safety cognition (i.e., safety climate), implicit safety cognition should strongly affect clear awareness.

By extending the social psychology theory described by Baron and Kenny [64], it is indicated that the interaction effect from explicit cognition and the initial external scenario would strongly affect the behavioural decision. However, through the mediation effect from clear cognition, and depending on the level of mediation, workers could redirect their own safety behaviours from being risky to behaving safely. This level of deviation would highly depend on the effectiveness of safety programs [69] and safety training [46] which could improve a positive safety climate.

### 5.2. Formation Mechanism of Construction Workers' Unsafe Behaviours

The analytic framework shown in Figure 2 can be further extended into the formation process based on the coded categories generated from Grounded Theory. Continued from Table 6, it is seen that unsafe behavioural outcomes are formed through one of the dynamic routes shown in Figure 3. Therefore, for any unsafe behaviour to occur, an external scenario that activates one individual desire, intervened by personal safety cognition, would drive the formation of the dominating motivation (i.e., benefit, stress, or emergency). It is stated in the Self-Determination Theory [70] that distinct types of motivations drive human behaviour. The motivation-driven behaviours could be performed to simply satisfy the innate psychological needs [71]. This need or desire is a necessary condition but not a sufficient condition for unsafe behaviour.

#### 5.2.1. Individual Desire Is Linked to Construction Workers' Unsafe Behaviour

The seven individual desires listed in Figure 3 reflect the need for workers and can be considered reasonable and regular human needs. Compared to other industries, the construction industry has more adverse and diverse site working conditions (e.g., hot to cool outdoor environments) and is deemed riskier in terms of health and safety. The subcontracted work in China's construction industry is more commonly based on a fixed amount, meaning that workers would gain the same payment by completing the given tasks. Hence, many workers prefer to save labour input and time to complete a fixed amount of work. Many workers migrate to urban areas of more economically developed regions in China in search of better-paying jobs. They may feel socially isolated and desire to be part of a social group. Therefore, they tend to follow peers' behaviours. The social outcome, defined by Man et al. [11], could be considered necessary for workers to enhance their image or status in the social system. External factors could also increase the probability of unsafe behaviour.

In many cases, construction projects in the urban areas of China are under a tight schedule to meet deadlines. As a result, it is common to see site workers committing to work overtime, including weekends. Therefore, any demands from the management that work should be completed earlier would result in more stress.

It should be a concern if workers' unsafe behaviours originate from their reasonable and rational human needs. It could be said that the unsafe behaviour is a by-product of the site work, meaning that the personal desire or conditions do not necessarily result in dangerous behaviours. It is not possible to eliminate these internal needs. What is more practical and feasible in safety management is to provide proper education by targeting workers' common personal needs, primarily to prevent them from conducting unsafe behaviours.

#### 5.2.2. The Effect of Safety Cognition on Unsafe Behaviours

Unsafe behaviours could allow an individual worker to achieve their own desired outcomes with positive or negative consequences (e.g., injuries). Therefore, before making a behavioural decision, each individual has to weigh the risk and benefits associated with a decision [72]. Indeed, construction workers would perceive it as worthwhile in trying a risky approach if there is a significant return from pursuing such unsafe behaviour. An immediate example is when having to save the lives of people in case of emergency or just simply to demonstrate their own “tough guy” syndrome [10] to peers and managers. Over time, and with sufficient safety awareness and perception of danger, workers may not decide to behave unsafely despite the benefits brought by the risky behaviour. The safety cognition system described in Figure 2 is insinuated as the mediated moderation factor between persona desire under specific external scenarios and the resulting motivation leading to a behavioural outcome. Furthermore, as workers gain more site experience and dexterity in perfecting their trade skills and routinely accomplish their work safely on a consistent level, safety awareness also increases at the same exponential knowledge of compliance. Therefore, it is more common to see workers being proactively and routinely safe by understanding how to conform to safety regulations. This is exhibited through increased avoidance of unsafe behaviours. Invariably, the antithesis is also actual. When workers behave unsafely, they are more likely to have sufficient safety awareness but exhibit low sensory understanding or anticipation of risks, hazards, and resulting harm or looming danger.

According to the Weber-Fechner Theory [73], human beings tend to become less sensitive to danger as their experience and skill grow, with less fear of danger. Almost befitting this theory is the expression that “familiarity breeds contempt” to warrant an effective response to compliance. There is virtually an infinite false sense of safety, qualified by unsafe behaviour. Expressing the same concern, Han et al. [37] compared their entry-level peers. They found that construction workers, in their middle careers, were more prone to underestimate the danger associated with site hazards. This conclusion is also prominent among workers who behave riskily but end up without encountering any negative consequences due to their behaviour. As for this group of workers, Kasperson et al. [74] have warned that the absence of negative experiences creates a false sense of safety. This predisposition to no harm leads to falsification of the actual benefits enshrined in full compliance, and conformance to safety standards has negative safety behaviour: workers become less sensitive to the potential risk-led dangers, are slow to be proactive, and become a danger to themselves and their fellow workers [74]. Workers' “fortunate” mindset would be strengthened under this scenario by relying on the superior supervision of foremen and sectional managers and the personal experience of co-workers. Holding the belief that accidents would not happen to themselves is what triggers or generates overconfidence and could deviate workers' safety perception and ability to improve their dexterities in their trade, and heighten the precipitation of perpetual acts of unsafe behaviours.

The factor of focusing on the end benefit (or benefit-driven) in addition to external pressure-induced desire is also a common trigger for unsafe behaviours. The explicit safety cognition (i.e., safety climate involving safe perception and safety awareness) plays a vital role as an intervening mediator to impact the behavioural decision. Under the proper mediation of safety cognition, workers could be equipped with sufficient safety awareness and sense of danger and hence not behaving unsafely. The occurrence of unsafe behaviour is not because of the internal personal desire but the failure of the mediated-moderation mechanism involving the safety cognition. Behaving unsafely but not causing accidents can strengthen and precipitate the “fortunate” behavioural syndrome mindset, thereby deteriorating the mediated moderation agent. Therefore, training workers with proper safety cognition to correct this bias of the “fortunate” attitude plays a key role.

#### 5.2.3. The Dynamic Mechanism in the Formation of Behavioural Decision

It is workers themselves who decide their behaviours. In other words, they can self-direct their motivation and behaviours. Despite the sufficient safety knowledge, site experience, and level of safety awareness, as well as the sense of danger due to unsafe behaviours, the internal desire (e.g., gaining more income) and external inducer (e.g., tight project schedule) may affect the behavioural decision-making according to the analytic framework in Figure 2. This dynamic mechanism also means that which motivation dominates can keep changing. For example, workers may feel uncertain in weighing the benefits and risks. The mediated moderation from safety cognition aims to guide workers towards safety-dominating motivation. The Protection Motivation Theory (PMT) [75] indicates that human beings naturally tend to protect themselves from danger if they can correctly sense the danger. According to PMT [75], the key is to nurture the proper sense of danger for workers, besides the education on safety awareness.

On the other hand, the Risk Homeostasis Theory [76] proposes that human beings opt to take more risks if they have a strong sense of safety. Workers may behave at different risk levels depending on the external conditions [77]. It happens that workers work in a more risky way because they believe that the superior safety devices can protect them from injuries, hence underestimating the site risks due to their unsafe behaviours. Klen [78] reported that workers behaved more carelessly in a riskier manner when equipped with protective equipment. However, this is not to deny the importance of wearing safety equipment. Instead, it highlights that the malfunctioning safety cognition system as the mediated moderation mechanism should be corrected to prevent unsafe behaviours.

#### 5.2.4. External Conditions That Affect Workers' Behavioural Decision

The adverse external conditions, such as high humidity and hot weather in summer construction and poor management commitment to safety, could exert a negative influence on workers' behaviours. This external influence cannot be ignored. The qualitative studies conducted in China's construction industry [10, 11] confirmed that workers are likely to be influenced by their peers' behaviours and the social values of their groups. If most other peers are working in an unsafe way, those minorities who behave in a standardized, safe manner may feel isolated and perhaps taken for granted. Safety management, therefore, plays a vital role in workers' behaviours. In some cases, workers quickly perform duties because they fear losing part of their income for failing to complete the job on time. Sometimes they must focus more on work efficiency to avoid being blamed by their managers.

Skinner's [79] theory indicates that timely and effective punishment for unsafe behaviour could prevent unsafe actions and motivations from reoccurring. However, insufficient safety monitoring and management system, which fails to handle safety violations timely and adequately, could motivate workers to behave unsafely and negatively affect their preestablished safety cognition system. A good safety management program is critical to forming a positive safety climate. The safety climate forms part of the safety cognition, which directly affects human behaviour [42]. A hostile site atmosphere (e.g., managers' emphasis on work efficiency and indifference to health and safety) could lead to the failure of the mediated moderation mechanism brought by the safety cognition system.

## 6. Conclusion

This study adopts a Grounded Theory approach to assist the investigation of the formation mechanism of construction workers' unsafe behaviour. Following site interviews with a total of 35 construction professionals, seven main categories were defined to describe workers' psychological needs or personal desires that led to unsafe behaviours. These seven categories, joint with the individual safety perception status, could lead to dangerous behaviours. The safety perception status measured by safety awareness and sense of danger could be incorporated into the explicit safety cognition. Individuals' psychological needs, activated by external scenarios and mediated by clear safety cognition, led to the behavioural outcome. The unsafe behaviour, according to the qualitative data obtained from this study, could be divided into proactive, passive, and reactive types, driven by different internal needs and external conditions on construction sites.

Following the data analysis adopting Grounded Theory, social psychology theories were applied in the context of construction safety behaviour. An analytic framework illustrating the formation of the behavioural decision was initiated by integrating the ideas of social cognitive psychology and safety cognition of construction workers. Social psychology highlighted the effects of two different factors (i.e., mediator and moderator) in affecting the behavioural outcome. The individual desire activated by an external scenario serves as one independent variable, which is one necessary but not sufficient condition for unsafe behaviours. The social psychology theories infer that construction workers' safety cognition system, consisting of implicit (e.g., safety knowledge and previous experience) and explicit (i.e., safety climate) cognitions, work as the mediated-moderation mechanism affecting the behavioural outcome. This is a dynamic mechanism involving workers' safety awareness and sense of danger, which intervene in their individual desires activated by external scenarios workers.

By integrating the results from Grounded Theory and the analytic framework, a further diagram was developed to describe the formation process of construction workers' unsafe behaviours. This diagram was discussed in depth in terms of four main aspects, namely, the original desires causing unsafe behaviours, the effects of safety cognition in unsafe behaviours, the dynamic mechanism informing behavioural decisions, and external conditions that affect workers' behavioural decisions. By applying a variety of social psychology theories, the diagram provides practical suggestions to properly guide construction workers' behaviours, including (1) an effective safety management program to provide effective training addressing workers' personal needs instead of ignoring or denying these needs or desires; (2) establishing a positive safety climate to develop workers' safety perception with sufficient safety awareness and sense of danger; and (3) periodic safety orientation targeting individuals' safety cognition system, not only for new workers but also especially for experienced workers. More specifically, many construction workers might not have received a university-level education, and alternative training manners other than toolbox meetings could be adopted, e.g., virtual reality-based site tour and video plays of safety hazards/accidents. Other measures to increase the safety awareness and sense of danger could include but be not limited to a proper frequency of safety inspection of workers on-site and an established management scheme between incentive and punishment to regulate safety behaviours.

One limitation of this current study is that all interviews were conducted in China's most economically active region, where construction projects are mostly under tight schedules with productivity highly stressed. More future work could be done to evaluate these defined categories from Grounded Theory in different regions or countries. The developed analytic framework and diagram can also be further applied in other regions or countries' construction sites. More future work can also investigate the effects of safety incentives and punishment policies in safety cognition development.

## Figures and Tables

**Table 1:** Descriptions of five general questions asked during the formal site interview.

No.	Question	Purpose
1	Could you describe the most unforgettable safety accident that you have heard of or experienced?	This is a starting and guiding question to motivate the interviewee to feel comfortable and become more engaged in the interview process by recalling their past site stories or experience. It also aims to spark the interviewee's thinking about safety.
2	What do you think about the cause of construction safety accidents? For example, are they more due to human mistakes or other reasons?	This commentary question aims to let the interviewee analyze the cause of accidents from the standpoint of a bystander who was not directly involved in the accident. It seeks to capture the core view of the interviewee as a nonbiased witness. It also guides the interviewee to pay attention to human factors related to unsafe behaviours gradually.
3	From your experience, were there some site behaviours of yourself that you feel could be dangerous?	This question serves as a transitional point aiming to shift the focus to the unsafe behaviour of the interviewee. In addition, it aims to let the interviewee realize their prior hazardous behaviour. The question was asked in a self-reflective approach, motivating the interviewee to recall and evaluate their dangerous behaviours (if any).
4	Why did you still decide to behave unsafely if you had realized the danger related to your unsafe behaviour?	This is a core question, continuing from the previous question to obtain the exact reasons that cause site workers to behave unsafely. In addition, it aims to capture site workers' psychological or mental status right before, during, and after conducting unsafe behaviours.
5	What unsafe behaviours do you see other people conduct? And what do you think are the main reasons why they behave unsafely?	This is a wrap-up question by shifting the focus from the interviewee themselves back to others. The interviewee answers the question again as a bystander to evaluate why peers behave unsafely. It is designed to acquire more in-depth thoughts related to unsafe behaviours. Also, because the initial question has been sensitive by asking the self-related dangerous behaviour, this question can relieve the nerve of the interviewee and allow the interviewee further to provide more information.

**Table 2:** Background information of the 35 interviewees.

No.	Age	Site experience (years)	Education level	Profession	Interview duration (min: sec)
1	55	30	Primary school	Concrete worker	27 : 42
2	32	13	Community college	Safety staff	24 : 55
3	50	30	High school	Project manager	43 : 00
4	40	8	Middle school	Crew foreman	30 : 59
5	30	5	Primary school	Electrical worker	28 : 27
6	25	8	High school	Ironworker	41 : 59
7	29	10	Middle school	General contractor's site employee worker	23 : 22
8	26	1	Master	Safety staff	32 : 21
9	23	1	Community college	General contractor's site employee	30 : 58
10	29	12	Community college	Crew foreman	42 : 46
11	27	2	Bachelor	Quality inspector	39 : 33
12	53	13	High school	Concrete worker	23 : 11
13	36	20	Middle school	Electrical worker	32 : 29
14	52	10	No education	Painter	25 : 14
15	48	20	High school	Office administrator	32 : 02
16	37	10	Primary school	Form worker	19 : 56
17	35	10	Bachelor	Quality inspector	37 : 48
18	35	10	Primary school	Ironworker	28 : 05
19	53	32	Middle school	Concrete worker	31 : 56
20	50	30	Middle school	Steelworker	27 : 07
21	60	10	Middle school	Site signal coordinator	27 : 17
22	50	20	Middle school	Steelworker	37 : 47
23	42	24	Middle school	Steelworker	18 : 09
24	50	30	Bachelor	Project manager	47 : 07
25	30	12	Middle school	Concrete worker	25 : 34
26	27	2	Bachelor	Technical support staff	26 : 58
27	25	1	Bachelor	Technical support staff	32 : 47
28	53	30	Primary school	Form worker	26 : 38
29	43	20	Middle school	Form worker	33 : 17
30	54	30	Primary school	Form worker	30 : 29
31	60	40	Primary school	Form worker	27 : 46
32	43	25	Middle school	Concrete worker	27 : 48
33	52	33	Primary school	Steelworker	30 : 05
34	30	7	High school	Form worker	35 : 24
35	45	23	Middle school	Steelworker	40 : 28

**Table 3:** List of concepts causing unsafe behaviours according to open coding.

Typical example(s) from interviewee's verbal messages	Concept	Frequency
“Sometimes, I prefer not to wear the safety belt when working at a height because that saves my effort.”	Saving efforts	34
“The main reason for some unsafe actions is to make more money in less time.”	Gaining more income	13
“I don't want to pay much attention to following these cumbersome steps. On the contrary, I want to ignore it and finish my work as soon as possible.”	Increasing productivity	10
“The reality is that workers must follow their managers' demands to complete the given tasks in a fast way.”	Being pressured	10
“Some guys feel that they are very skilled to perform tasks.”	Being overconfident	10
“When the schedule is tight, the boss asks us to work overtime and maintain the efficiency.”	Meeting schedule requirements	9
“The main reason for unsafe behaviour is to save time.”	Saving time	7
“They think it is their freedom to not wear a hard hat”; “If the manager wants them to wear the hard hat, they may still not fasten the tie of the hat.”	Being unwilling to be regulated	6
“The weather in the summer is hot, and not wearing the protective equipment makes me feel more comfortable.”	Feeling more comfortable	6
“The manager asks us to do things fast and complete the tasks quickly; otherwise, we could lose our job.”	Following the manager's demand	5
“Older and more experienced folks can do things fast without risks”; “Older guys think they know things well enough and do not need to follow the safety education.”	Following personal experience	5
“Safety accidents occurred very rarely on sites where I work during my past five years' career. I don't think I would be that unlucky.” “They think they are lucky enough to be accident-free by not wearing the safety boots.”	Holding a “fortunate” mindfulness	5
“Some guys are rebellious and do not want to follow what their managers say.”	Being against the safety demand	4
“Sometimes we want to relax a bit after considerable experience site work, without realizing site safety risks.”	Relaxing	4
“A crew foreman just learned that he was about to be fired. He hammered a nail into an electrical cable to show his anger before he left. Later a fire accident happened when the cable was powered.”	Venting the negative emotion	3
“Some younger workers behave unsafely to show their own ‘tough-guy' image.”	Showing off self-capability	3
“Two different woodwork teams needed tower cranes to transport materials, and conflicts happened. Both teams did not want to calm down. One of them broke the electrical cable of the tower crane on purpose.”	Escaping responsibilities	3
“Two apprentices were working together with their mentor. One of the apprentices was trying to impress the mentor during the installation of scaffolding when the mentor was not around for a moment. He climbed into the scaffold trying to operate, but later he fell off from scaffolding and was injured.”	Demonstrating own skills	3
“This type of part-time guys deliberately waste time, not doing jobs safely.”	Dawdling	3
“I just want to operate the machine myself to see how it works.”	Satisfying curiosity	2
“The wastes from saw-cuts fell on flammable things, but workers are hungry and want to have their lunch. So they ignore that.”	Being anxious to finish work	2
“Some guys walk along the steel pipes without fall protection just to feel excited.”	Seeking excitement	2
“Arguments may happen between different trade teams because of interests of conflict.”	Defending for the benefit of their team	2
“If everyone else is working unsafely to complete work on time, I will follow them.”	Following peer behaviours	2

*Note*. Table 1 does not cover coding concepts only mentioned once by the 30 interviewees. They include maintaining self-esteem, meeting self-vanity, helping others in an emergency, and horseplaying.

**Table 4:** Initial categories summarized through open coding.

Initially coded category	Defined feature	Mentioned by number of interviewees
Saving effort	Some workers consider the standard practice cumbersome and desire that the task be completed in a relatively more straightforward manner, such as throwing tools to deliver them between peers, crossing the safety fence, installing the scaffolding without fall protection, and randomly placing tools/materials on-site for the sake of convenience.	13
Gaining more income	Some workers perform their jobs with sicknesses or fatigue to gain more income or bonus.	11
Being overconfident	Some workers underestimate the risk of their behaviours and overestimate their capability to control risks.	8
Fearing losing jobs	Some workers opt to do unsafe work if their managers require them to finish on time or catch up with the construction schedule.	7
Meeting the scheduling needs	Some workers work overtime under fatigue to catch up with the scheduling requirements.	7
Saving time	Some workers skip steps in the standard construction workflow by violating safety regulations to save time. Some crew miss the safety education just to gain more time performing site duties.	7
Pursuing work efficiency	Some workers desire to complete duties in less time and behave in more risky ways, such as dropping the concrete formwork and scaffolding fasteners and manually carrying heavy items on-site.	5
Coping with safety inspection	Some site workers conceal or temporarily hide site items that do not comply with safety regulations to cope with periodic safety inspections from authorities or third parties.	5
Relying on personal experience	Some workers highly rely on their past site experience to judge the risk of their behaviour, and some unsafe behaviours may occur due to their long-term risk-taking habits or experience.	5
Holding the “being lucky” mindset	Some workers underestimate the safety issues, being even more “optimistic” towards safety if their past violation of safety rules did not cause accidents. However, they also believe that they would not be unfortunate to be involved in accidents.	5
Seeking comfort	Workers feel that wearing a hard hat, fastening safety belts, or using other safety protective equipment would make them even more uncomfortable in the hot weather.	4
Following the manager's demands	Workers feel unable to resist the commands of their crew leader. They feel obliged to perform risky duties and require high professional skills beyond their capability. As a result, they violate safety regulations to prevent project delays.	4
Resisting being regulated	When facing the blame or punishment for a safety violation, some workers turn out to be rebellious and feel unfairly treated and desire to continue their unsafe behaviours.	4
Reducing fatigue or pressure	Labour-intense duties and uncomfortable site conditions (noisy and hot) make workers feel exhausted and drive them irritable. As a result, some may smoke, snore, or even drink alcohol on-site.	3
Expressing emotions	Some workers are annoyed and angered for several reasons, including being rudely treated or blamed by site management personnel, family issues, and fairly punishment; as a result, they behave on purpose against the demands of their managers as a way to express their anger, such as by not attending mandatory safety education.	3
Escaping responsibilities	Some site workers lack a sense of responsibility, feel reluctant to be part of the site inspection, hide unsafe conditions or not report safety accidents, or blame others for the aroused safety issues.	3
Revenging	Conflicts may happen between workers and management personnel or among different trades. Some site workers seek opportunities to revenge by damaging others' work outputs.	3
Demonstrating capability	Some workers desire to impress their managers and demonstrate their capabilities by behaving differently from their peers.	3
Showing a “tough guy” image	Some workers desire to show their “tough guy” image to their peers and line managers. They do not follow management guidelines and pretend that they are experienced and know what they would perform, resulting in risky behaviours and safety violations.	2
Idling	Some part-time workers do not care about safety or perform their high-quality jobs. Instead, they mainly focus on gaining their daily income by spending their time on-site.	2
Hurrying to complete work	Once upon completing their daily duties, some workers are anxious to return home by finishing the last piece of work in a hurry and further cause accidents, e.g., falling from height.	2
Satisfying curiosity	Some workers feel curious and operate equipment (e.g., tower crane) without proper training.	1
Seeking excitement	Some workers behave unsafely by jumping on-site, throwing tools to deliver them, horseplaying, or playing in dark and confined spaces (e.g., basement, culverts).	1
Following peers	Although they do not agree with some unsafe actions conducted by co-workers, some workers still decide to follow their peers' unsafe behaviours to be social in their workgroup.	1
Maintaining self-esteem	When some workers feel insulted on-site, they may react in an extreme or unsafe way to defend their self-esteem.	1
Enhancing self-vanity	Some workers perform risky tasks beyond their control to gain praise from others.	1
Disturbing other trade teams' work	Some workers use the tools or equipment from other trade groups without permission just to benefit their group.	1
Reacting in emergency	Some workers, although without sufficient safety training, react in an emergency by trying to help others in danger.	1
Avoiding being monitored	Some workers deliberately hide from their managers to act unsafely.	1
Being distracted	Some workers are thinking of other nonwork-related things when working on-site.	1

**Table 5:** Coded main categories linked to initially coded categories.

Main category	Initially coded categories	The intention or motivation of unsafe behaviours
Reducing physical work	Saving effort; coping with safety inspection; seeking comfort; idling	Minimizing discomfort at work; reducing labour input
Reducing time input	Saving time; pursuing work efficiency; hurrying to complete work	Reducing the time spent on performing duties
Meeting internal desires	Being overconfident; relying on personal experience; holding the “being lucky” mindset; resisting being regulated; expressing emotions; escaping responsibilities; revenging; showing a “tough guy” image; demonstrating capability; satisfying curiosity; seeking excitement; enhancing self-vanity; maintaining self-esteem; avoiding being monitored; being distracted	Meeting a specific psychological need or desire; seeking a specific type of internal satisfaction (e.g., curiosity)
Relieving stress at work	Fearing of losing jobs; meeting the scheduling needs; following the manager's demands; reducing fatigue or pressure	Relieving stress driven by a specific type of external scenario
Increasing income	Gaining more income	Gaining more income from work
Being part of the team	Following peers; disturbing other trade teams' work	Demonstrating self-conformance to the own social or workgroup
Helping others	Reacting in emergency	Saving others from danger despite self-incompetency

**Table 6:** Internal connections for each coded category, individual status of safety perception, and the corresponding behavioural outcome.

Connection among each coded category, individual safety perception status, and potential behavioural outcome	Definition
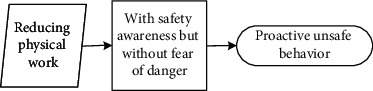	Before conducting unsafe behaviour, workers have a certain understanding of the risk involved in their unsafe actions. However, they either underestimate the danger, hold the “fortunate” mindset believing that accidents are not likely to occur to themselves, or desire to seek convenience or comfort.
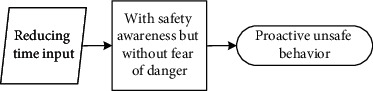	Before conducting unsafe behaviour, workers have a certain understanding of the risk involved in their unsafe actions. However, they underestimate the danger or hold the belief that they should not be that unlucky to become victims of accidents. They also care more about completing site work in less time.
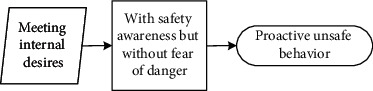	Before conducting unsafe behaviour, workers thoroughly understand the risk involved in their unsafe actions. But they also desire to satisfy specific internal needs by ignoring or underestimating the danger and violating safety rules.
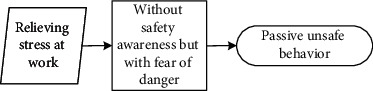	Before conducting unsafe behaviour, workers have a certain degree of fear of the potential danger. However, they still run dangerous behaviours due to stress caused by external scenarios and improper safety awareness due to a lack of professional knowledge.
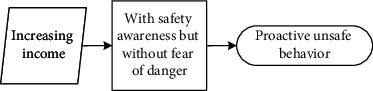	Before conducting unsafe behaviour, workers have a specific understanding of the risk involved in their unsafe actions. However, they underestimate the danger or hold the belief that they should not be that unlucky to become victims of accidents. They also care more about earning more rather than safety.
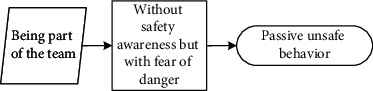	Before conducting unsafe behaviour, workers have a certain degree of fear of the potential danger. But they lack sufficient knowledge of the risk related to their hazardous behaviour. Peers' unsafe behaviour would encourage them more to behave unsafely in order to show themselves as part of the team.
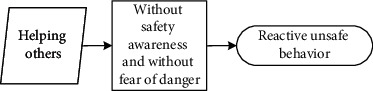	Workers may not have sufficient safety knowledge of risks, and nor do they fear the danger involved. They desire to help other people on-site, under unexpected conditions or emergencies.

## Data Availability

Data generated or analyzed during the study are available from the corresponding author by request.
